# Independent Prognostic Value of *BRAF V600E* for Recurrence in Papillary Thyroid Carcinoma: A Systematic Review and Meta-Analysis

**DOI:** 10.3390/cancers18142299

**Published:** 2026-07-17

**Authors:** Sandeep Kumar Parvathareddy, Zeeshan Qadri, Maha Al-Rasheed, Saud Azam, Nabil Siraj, Saif S. Al-Sobhi, Fouad Al-Dayel, Khawla S. Al-Kuraya

**Affiliations:** 1 Human Cancer Genomic Research, King Faisal Specialist Hospital and Research Centre, P.O. Box 3354, Riyadh 11211, Saudi Arabia; psandeepkumar@kfshrc.edu.sa (S.K.P.); sqadri96@kfshrc.edu.sa (Z.Q.); mrasheed@kfshrc.edu.sa (M.A.-R.); sjeelani@kfshrc.edu.sa (S.A.); nsiraj@kfshrc.edu.sa (N.S.); 2 Department of Surgery, King Faisal Specialist Hospital and Research Centre, P.O. Box 3354, Riyadh 11211, Saudi Arabia; sobhi@kfshrc.edu.sa; 3 Department of Pathology, King Faisal Specialist Hospital and Research Centre, P.O. Box 3354, Riyadh 11211, Saudi Arabia; dayelf@kfshrc.edu.sa

**Keywords:** papillary thyroid carcinoma, *BRAF V600E*, recurrence, systematic review, meta-analysis

## Abstract

**Background:** The independent prognostic value of *BRAF V600E* in papillary thyroid carcinoma (PTC) remains uncertain. Current risk stratification schemes incorporate *BRAF* inconsistently, reflecting uncertainty about its independent association with recurrence. A systematic review and meta-analysis were conducted to evaluate the association between *BRAF V600E* and recurrence-related outcomes in PTC. **Methods:** PubMed and Embase were searched for observational cohort studies reporting recurrence, recurrence-free survival, or disease-free survival according to *BRAF* status and providing multivariate-adjusted hazard ratios (HRs). Primary analysis included studies in which multivariate models adjusted for tumor size or American Joint Committee on Cancer (AJCC) tumor (T) category, whereas sensitivity analysis included all eligible multivariate studies irrespective of tumor size adjustment. Random-effects models were used to pool HRs with 95% confidence intervals (CIs), and heterogeneity was quantified with I^2^. Thirty-five cohort studies (*n* = 14,982 patients) met the inclusion criteria. **Results:** In the primary analysis of 27 studies adjusting for tumor size or T category, *BRAF V600E* was associated with a 58% higher risk of recurrence (pooled HR 1.58, 95% CI 1.25–1.99; I^2^ = 56.2%). In the sensitivity analysis, results remained consistent (pooled HR 1.40, 95% CI 1.11–1.76; I^2^ = 64.5%). The funnel plot and Egger’s regression test did not indicate substantial publication bias. **Conclusions:** Overall, *BRAF V600E* was associated with a modest but statistically significant increase in recurrence risk in PTC after adjustment for tumor size or T category. However, given the heterogeneity of included studies and variability in multivariable adjustment, these findings should be interpreted as supporting *BRAF V600E* as a complementary rather than a standalone prognostic marker.

## Simple Summary

Papillary thyroid cancer is the most common type of thyroid cancer and usually has an excellent prognosis. However, some patients experience cancer recurrence after treatment, making accurate risk assessment important for guiding follow-up care. A genetic alteration known as *BRAF V600E* has been widely studied as a possible predictor of recurrence, but previous studies have reported conflicting results. In this study, we systematically reviewed and combined data from more than 14,000 patients across 35 published studies to determine whether this genetic change independently increases the risk of recurrence. Our findings show that patients whose tumors carry the *BRAF V600E* mutation have a modestly higher risk of recurrence, even after accounting for important clinical factors such as tumor size. These results support the use of *BRAF* status as an additional tool for risk assessment while emphasizing that established clinical features remain the most important predictors of outcome.

## 1. Introduction

Papillary thyroid carcinoma (PTC) is the most common endocrine malignancy and is generally associated with excellent long-term outcomes [1,2]. Nevertheless, 10–30% of patients develop locoregional or distant recurrence, leading to increased healthcare utilization and reduced quality of life [3,4,5]. Accurate postoperative risk stratification is therefore essential to tailor surveillance strategies and adjuvant therapy.

The *BRAF V600E* mutation is the most prevalent oncogenic driver in PTC, occurring in approximately 45–60% of cases worldwide [6,7,8]. According to The Cancer Genome Atlas (TCGA), most papillary thyroid carcinomas harboring the *BRAF V600E* mutation belong to the *BRAF*-like molecular subtype, whereas tumors driven by *RAS* mutations are classified as *RAS*-like. This molecular classification has subsequently been incorporated into the 5th edition of the WHO Classification of Endocrine and Neuroendocrine Tumours and provides additional biological context for the clinical behavior associated with *BRAF*-mutant PTC [9]. Activation of the MAPK pathway by *BRAF V600E* promotes thyroid cell dedifferentiation through suppression of thyroid-specific genes, including the sodium-iodide symporter (NIS) and thyroid peroxidase (TPO). Consequently, *BRAF*-mutant tumors frequently demonstrate reduced radioiodine avidity, which has important implications for postoperative management [10]. These biological effects provide a plausible rationale for why *BRAF V600E* has been associated with adverse clinicopathologic features in many observational studies. However, biological plausibility alone does not establish independent prognostic value. In PTC, recurrence risk is strongly influenced by tumor size, extrathyroidal extension, nodal burden, surgical extent, radioiodine use, and duration of follow-up [11,12,13,14,15]. Because *BRAF V600E* is itself associated with several of these adverse features, unadjusted analyses may overestimate its true contribution to recurrence. Therefore, determining whether *BRAF V600E* retains prognostic significance after adjustment for established clinicopathologic factors remains clinically important.

Early studies demonstrated strong associations between *BRAF V600E* and aggressive tumor characteristics, including extrathyroidal extension, lymph node metastasis, and advanced TNM stage [16,17,18]. Several large multicenter cohorts also suggested that *BRAF V600E* showed a modest independent association with recurrence after adjustment for clinicopathologic variables [19,20], prompting proposals to incorporate *BRAF* status into risk stratification frameworks [21]. However, other studies—including large population-based analyses—failed to demonstrate an independent prognostic impact of *BRAF* once tumor size, lymph node status and age were controlled for [22,23,24,25]. Recent data, including our own large institutional study, further showed that tumor size remained the dominant predictor of recurrence, while *BRAF V600E* lost significance in multivariate models [22,26]. These conflicting results contribute to persistent uncertainty in clinical guidelines regarding whether *BRAF* status should influence postoperative decision-making [27]. Recent meta-analyses have evaluated the prognostic role of *BRAF V600E* in PTC; however, many pooled heterogeneous outcomes did not consistently distinguish between adjusted and unadjusted estimates or prioritize models accounting for tumor size [28]. This distinction is particularly relevant in contemporary thyroid cancer management, where molecular testing is increasingly incorporated into diagnostic and postoperative workflows. A molecular marker is most clinically useful when it adds information beyond routinely available histopathologic variables rather than merely reflecting their presence. For *BRAF V600E*, this distinction remains unresolved because published studies have differed in outcome definitions, follow-up duration, adjustment covariates, and whether recurrence, persistence, recurrence-free survival, or disease-free survival were analyzed as separate endpoints. A focused synthesis of multivariable-adjusted time-to-event estimates is therefore needed to clarify the magnitude and limitations of the *BRAF*-associated recurrence signal.

Accordingly, the independent contribution of *BRAF V600E* to recurrence risk remains insufficiently defined. To address this gap, we conducted a systematic review and meta-analysis restricted to multivariate-adjusted hazard ratios for recurrence-related outcomes, with a primary analysis focused on studies adjusting for tumor size or T category. Our objective was to determine whether *BRAF V600E* provides independent prognostic value beyond established clinicopathologic factors—particularly tumor size.

## 2. Materials and Methods

### 2.1. Study Design

This systematic review and meta-analysis evaluated the association between *BRAF V600E* and recurrence-related outcomes in patients with PTC. Reporting followed PRISMA 2020 guidelines for meta-analyses of observational studies [29] (checklist provided as Supplementary Table S1). Prospective protocol registration (e.g., PROSPERO) was not performed. Ethical approval was not required, as this study was a systematic review and meta-analysis of previously published data.

### 2.2. Literature Search

A comprehensive search of PubMed and Embase was performed. Three reviewers independently performed study selection and data extraction; discrepancies were resolved by consensus.

The PubMed search strategy was as follows:

(“papillary thyroid carcinoma ”[Title/Abstract] OR “PTC” [Title/Abstract] OR “differentiated thyroid cancer” [Title/Abstract] OR “thyroid carcinoma” [Title/Abstract]) AND (“*BRAF*” [Title/Abstract] OR “*BRAF V600E*” [Title/Abstract] OR “*BRAF* mutation” [Title/Abstract]) AND (“recurrence” [Title/Abstract] OR “disease-free survival (DFS)” [Title/Abstract] OR “DFS” [Title/Abstract] OR “outcome” [Title/Abstract]) AND (“hazard ratio(HR)” [Title/Abstract] OR “HR” [Title/Abstract] OR “multivariate” [Title/Abstract] OR “Cox” [Title/Abstract])

The Embase strategy was as follows:

(‘papillary thyroid carcinoma’:ab,ti OR ‘differentiated thyroid cancer’:ab,ti OR ‘thyroid carcinoma’:ab,ti) AND (‘braf’:ab,ti OR ‘braf v600e’:ab,ti OR ‘braf mutation’:ab,ti) AND (‘recurrence’:ab,ti OR ‘disease free survival’:ab,ti OR ‘dfs’:ab,ti OR ‘outcome’:ab,ti) AND (‘hazard ratio’:ab,ti OR ‘multivariate’:ab,ti OR ‘cox’:ab,ti)

Searches were limited to human studies, studies in the English language, and original research. Reference lists of included studies were screened to identify any additional eligible articles.

Before submission of the revised manuscript, the literature search was updated on 02 July 2026 using a broader search strategy that removed the mandatory requirement for the terms ‘hazard ratio’, ‘multivariate’, or ‘Cox’ in the title or abstract. The updated search identified six additional eligible studies, which were incorporated into the final quantitative synthesis, yielding a total of 35 eligible studies.

### 2.3. Eligibility Criteria

Eligibility criteria followed the PICOS framework. Studies were included if they met all of the following:

1. Adult patients with histologically confirmed PTC or extractable PTC-specific data;
2. *BRAF V600E* mutation status reported;
3. At least one recurrence-related outcome (recurrence, recurrence-free survival, disease-free survival, or structurally persistent disease);
4. Multivariate-adjusted hazard ratio (HR) with 95% confidence interval (CI) comparing *BRAF*-mutant vs. wild-type tumors;
5. Observational cohort design (retrospective).

Exclusion criteria:

- Case reports, case series, reviews, editorials, or conference abstracts;
- Studies without recurrence-related outcomes or without multivariate HRs;
- Duplicate cohorts (most complete or most recent dataset retained).

### 2.4. Study Selection and Data Extraction

Three reviewers independently screened titles/abstracts and full texts; discrepancies were resolved by consensus. Extracted data included the following:

- First author, publication year, country;
- Study design, total sample size, *BRAF*-positive/negative counts;
- Outcome type;
- Multivariate HR and 95% CI;
- Covariates included in the multivariate model (whether tumor size/T category, age, and lymph node status were adjusted for);
- AJCC edition and median follow-up duration.

Only multivariate HRs were used for the analysis. When multiple models existed, the model adjusting for tumor size or T category was preferentially extracted. This approach was chosen to minimize confounding arising from differences in baseline tumor characteristics and to better estimate the independent prognostic contribution of *BRAF V600E*. Because recurrence in papillary thyroid carcinoma is strongly influenced by established clinicopathologic variables, including tumor size, lymph node involvement, and extrathyroidal extension, analyses based on multivariable-adjusted hazard ratios were considered more appropriate than unadjusted estimates for evaluating the incremental prognostic value of *BRAF V600E* across heterogeneous observational cohorts.

Potentially overlapping cohorts were assessed by comparing study institution, recruitment period, author group, sample size, and patient characteristics. When overlap was identified, only the most complete or most appropriate dataset was retained.

### 2.5. Risk of Bias Assessment

Risk of bias for each study was assessed using the Newcastle–Ottawa Scale (NOS) for cohort studies and graded as low, moderate, or high risk across selection, comparability, and outcome domains.

### 2.6. Outcomes

The primary endpoint was a recurrence-related time-to-event outcome. A predefined endpoint hierarchy was used during data extraction. Structural recurrence was prioritized whenever reported, followed by recurrence-free survival, persistent structural disease, disease-free survival, and progression-free survival. Although these outcomes are related, they represent different clinical constructs. Therefore, subgroup analyses were performed to evaluate recurrence-related outcomes and disease-free survival separately.

### 2.7. Primary and Sensitivity Analyses

The primary analysis included studies in which multivariate models were adjusted for tumor size or T category, ensuring independent assessment of *BRAF V600E*.

A sensitivity analysis included all eligible multivariate studies regardless of tumor size adjustment.

### 2.8. Statistical Analysis

Meta-analysis was performed using R version 4.5.1 (R Foundation for Statistical Computing, Vienna, Austria). Random-effects models were applied using the meta package (version 7.0-0), employing the metagen() function to pool log HRs with inverse-variance weighting. Funnel plots were generated using the metafor package (version 4.2-0). Publication bias was assessed using funnel plots and Egger’s regression test as well as the trim-and-fill method. Heterogeneity was assessed using Cochran’s Q and I^2^ statistics. Statistical significance was defined as two-sided *p* < 0.05.

## 3. Results

### 3.1. Study Selection

**Figure 1:**
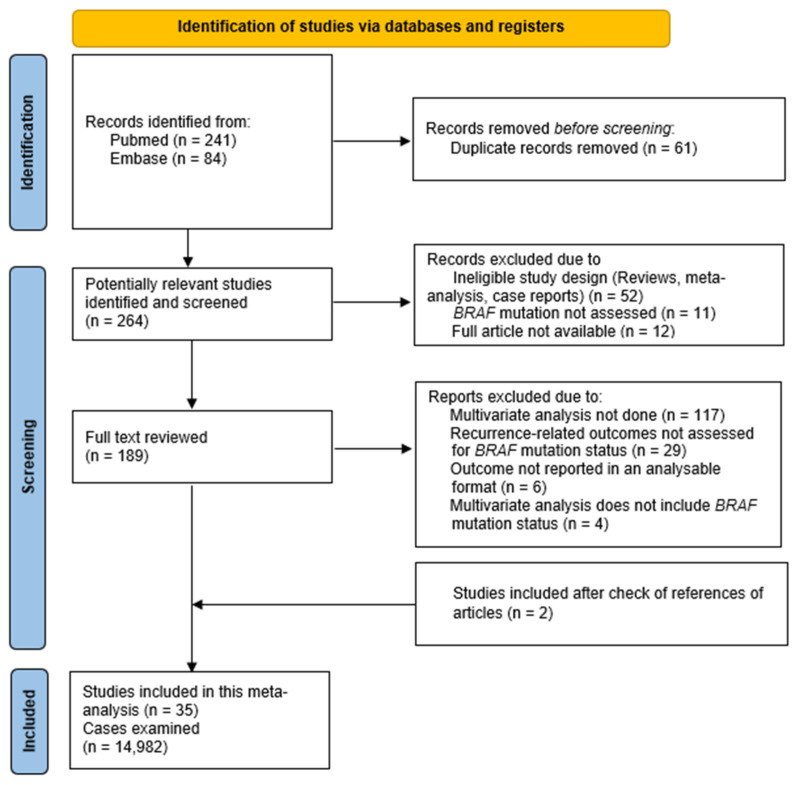
PRISMA 2020 flow diagram for new systematic reviews, which included searches of databases and registers only. From Page et al. [29] (for more information, visit http://www.prisma-statement.org/ (accessed on 10 February 2026).

The final literature search identified 325 records (PubMed: 241; Embase: 84). Initial search identified 177 studies and following the expanded literature search performed on 2 July 2026, an additional 148 records were identified. After removal of duplicates, title/abstract screening, and full-text assessment, 35 eligible studies were included in the final quantitative synthesis (during reassessment, two duplicate cohorts were identified and removed before the final meta-analysis). The complete study selection process is summarized in Figure 1.

### 3.2. Study Characteristics

All included articles were published between 2005 and 2026, comprising 14,982 patients with PTC. Sample sizes ranged from 41 to 2099 patients. All included studies were retrospective observational cohort studies and reported recurrence, recurrence-free survival, or disease-free survival as the recurrence-related endpoint. The median follow-up duration, when reported, ranged from 15 to 180 months.

Twenty-seven studies were adjusted for tumor size or T category in multivariate models, while the remainder included other covariates but did not explicitly adjust for tumor size. Adjustments for lymph node status and age varied across studies. Key characteristics of all included studies are summarized in Table 1.

Using the Newcastle–Ottawa Scale, overall study quality was predominantly high (scores 7–9). To improve transparency regarding multivariate adjustment, Supplementary Table S2 summarizes the covariates included in each multivariate model across all eligible studies as well as the NOS score of each study.

### 3.3. Primary Meta-Analysis

**Figure 2:**
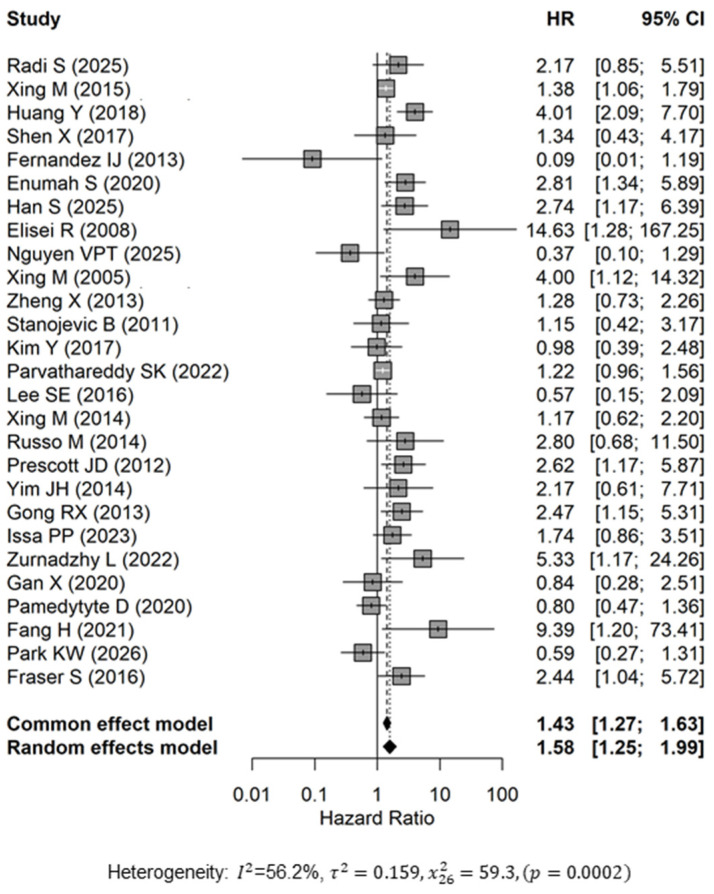
**Forest plot (primary analysis):** Forest plot showing the pooled hazard ratios (HRs) and 95% confidence intervals (CIs) for recurrence among the 27 studies ([32], [19], [45], [46], [53], [40], [33], [59], [20], [60], [54], [58], [47], [36], [49], [4], [51], [56], [52], [55], [24], [37], [42], [43], [39], [30] and [50], respectively) adjusting for tumor size or T category. Heterogeneity is summarized using the I^2^ statistic. The solid squares represent the study-specific HR estimates, with the size of each square proportional to the weight assigned to the study in the meta-analysis. Horizontal lines indicate the corresponding 95% CIs. The vertical solid line at HR = 1 represents the line of no effect. The dashed vertical line indicates the pooled effect estimate. Diamonds represent the pooled HRs, with the center of each diamond indicating the summary estimate and the width representing the 95% CI. Results are presented for both the common-effect (fixed-effect) and random-effects models.

Across the 27 studies adjusting for tumor size or T category, *BRAF V600E* was associated with a significantly increased risk of recurrence (pooled HR 1.58, 95% CI 1.25–1.99), with moderate heterogeneity (I^2^ = 56.2%) (Figure 2).

### 3.4. Sensitivity Analysis

**Figure 3:**
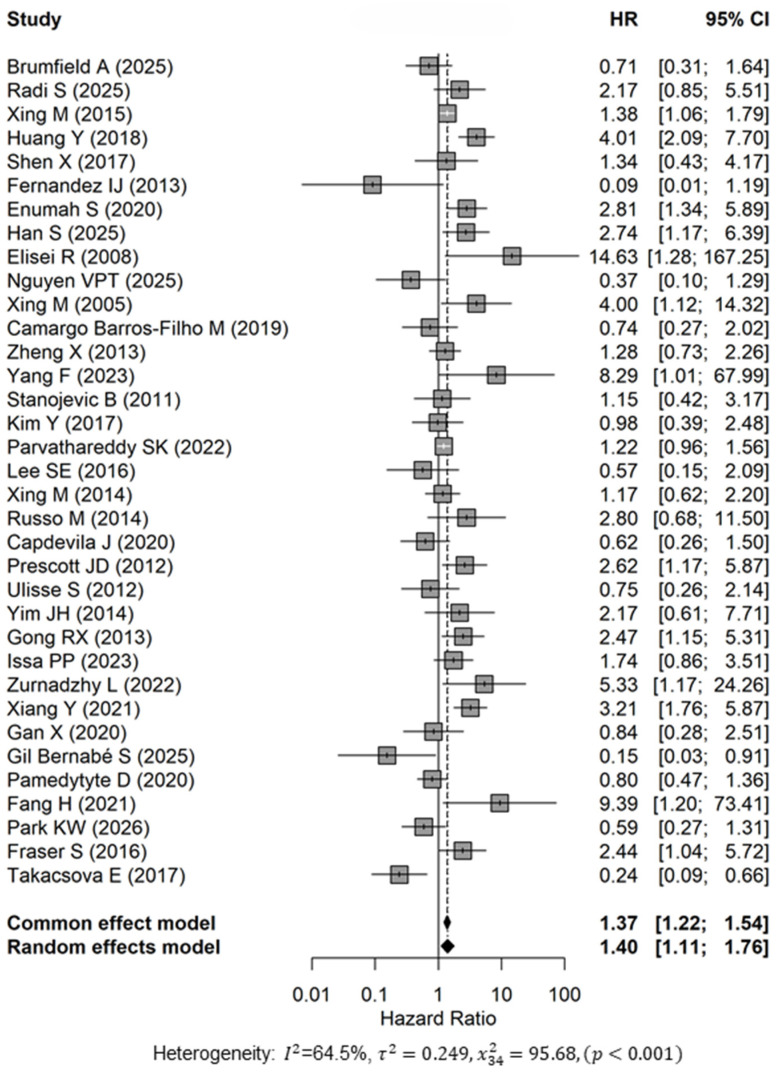
**Forest plot (sensitivity analysis):** Forest plot showing the pooled hazard ratios (HRs) and 95% confidence intervals (CIs) for recurrence among the 35 studies (irrespective of whether they were adjusted for tumor size/T category or not) ([31], [32], [19], [45], [46], [53], [40], [33], [59], [20], [60], [44], [54], [35], [58], [47], [36], [49], [4], [51], [41], [56], [57], [52], [55], [24], [37], [38], [42], [34], [43], [39], [30], [50] and [48], respectively). Heterogeneity is summarized using the I^2^ statistic. The solid squares represent the study-specific HR estimates, with the size of each square proportional to the weight assigned to the study in the meta-analysis. Horizontal lines indicate the corresponding 95% confidence intervals (CIs). The vertical solid line at HR = 1 represents the line of no effect. The dashed vertical line indicates the pooled effect estimate. Diamonds represent the pooled HRs, with the center of each diamond indicating the summary estimate and the width representing the 95% CI. Results are presented for both the common-effect (fixed-effect) and random-effects models.

When all 35 eligible multivariate studies were included, the association remained significant and consistent (pooled HR 1.40, 95% CI 1.11–1.76; I^2^ = 64.5%) (Figure 3). Leave-one-out influence diagnostics confirmed the robustness of the results. Subgroup analysis based on outcomes showed that the significant association was primarily driven by a strong effect on recurrence (pooled HR 1.53, 95% CI 1.17–2.01; I^2^ = 61.2%) (Supplementary Figure S1), while no statistically significant association was observed for DFS (pooled HR 1.07, 95% CI 0.66–1.74; I^2^ = 67.0%) (Supplementary Figure S2), with moderate between-study heterogeneity. These findings indicate that the overall association was primarily driven by studies reporting structural recurrence or recurrence-related outcomes, whereas no statistically significant independent association was observed for disease-free survival.

### 3.5. Meta-Regression Analysis for Tumor Size and Lymph Node Metastasis

Meta-regression analysis was performed to explore whether adjustment for tumor size and lymph node status influenced the observed effect estimates. Neither tumor size (*p* = 0.081) nor lymph node adjustment (*p* = 0.729) demonstrated a statistically significant association with the outcome of interest. Furthermore, the model explained none of the between-study heterogeneity (R^2^ = 0%). However, because the meta-regression was based on study-level binary indicators of adjustment rather than patient-level covariates, these findings should be interpreted cautiously and should not be considered evidence that tumor size or lymph node status are unimportant prognostic factors.

### 3.6. Publication Bias

**Figure 4:**
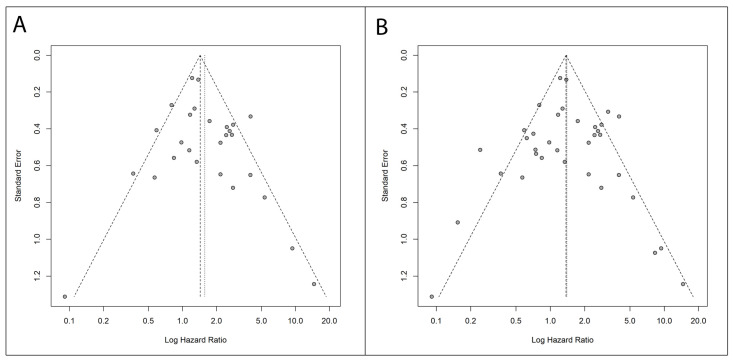
**Funnel plot for publication bias:** The funnel plot displays log hazard ratios versus their standard errors for the included studies. Each dot represents an individual study included in the meta-analysis, plotted according to its effect estimate (log hazard ratio) on the x-axis and its standard error on the y-axis. Visual inspection shows a roughly symmetrical distribution around the pooled effect estimate, suggesting no substantial publication bias for studies included either in the (**A**) primary analysis or (**B**) sensitivity analysis.

Visual evaluation of funnel plots (Figure 4) and Egger’s regression test (*p* = 0.838) showed no evidence of small-study effects. As a complementary sensitivity assessment, trim-and-fill analysis imputed one potentially missing study. The adjusted pooled estimate remained statistically significant (HR = 1.37, 95% CI = 1.08–1.73), supporting the stability of the overall finding (Supplementary Figure S3).

## 4. Discussion

The prognostic significance of *BRAF V600E* in PTC remains a subject of ongoing debate despite extensive research [27,28]. In this systematic review and meta-analysis restricted to studies reporting multivariate-adjusted effect estimates, *BRAF V600E* was associated with a modest but statistically significant increase in recurrence risk, even in models controlling for tumor size or T category. When all eligible multivariate studies were included irrespective of tumor size adjustment, the association persisted with similar effect size and precision. These findings suggest that *BRAF V600E* is associated with a modest increase in recurrence risk after multivariable adjustment, although the magnitude of this association should be interpreted in the context of heterogeneous study designs and adjustment strategies. After expansion of the literature search, inclusion of six additional eligible studies, and removal of two duplicate cohorts, the overall direction and statistical significance of the main findings remained unchanged.

An important implication of these findings is the distinction between statistical significance and clinical utility. Although *BRAF V600E* remained significantly associated with recurrence after multivariable adjustment, the observed effect size was modest. This suggests that *BRAF V600E* should not be interpreted as an isolated determinant of prognosis but rather as one component of a broader clinicopathologic and molecular risk profile. Contemporary risk stratification increasingly emphasizes integrated models that combine conventional histopathologic variables with molecular alterations to improve individualized patient assessment. Accordingly, *BRAF V600E* should be interpreted as refining, rather than redefining, postoperative risk assessment.

Although the included studies reported related recurrence endpoints, these outcomes are not identical and differ in clinical definition. Therefore, we interpreted the pooled estimate alongside outcome-stratified analyses. Subgroup analysis showed that the overall effect is primarily driven by recurrence-related outcomes, where the pooled hazard ratio reached 1.53 and remained statistically significant with moderate precision. This suggests that the exposure may be more strongly linked to biological mechanisms underlying tumor recurrence rather than broader survival endpoints. In contrast, no statistically significant association was observed for DFS, as evidenced by the subgroup’s confidence interval crossing one. This finding likely reflects both the limited number of available studies and heterogeneity in the definition of disease-free survival across cohorts, which differs from structural recurrence and may capture a broader spectrum of clinical outcomes. Accordingly, the results should be interpreted as demonstrating the strongest and most consistent association for structural recurrence rather than implying an equivalent effect across all recurrence-related endpoints. Our findings suggest that *BRAF V600E* is associated with a modest increase in the risk of structural recurrence in PTC, whereas the evidence for disease-free survival remains less consistent.

Several large studies have demonstrated that *BRAF V600E* independently predicts recurrence after adjustment for tumor size, lymph node status, and extrathyroidal extension [19,28,40,61], supporting integration into postoperative stratification systems. Conversely, other cohorts have failed to demonstrate independent prognostic value for *BRAF V600E* once traditional risk variables are accounted for [22,23,24,25,31]. Recent institutional and population-based analyses have similarly shown that tumor size remains the dominant determinant of recurrence, while *BRAF V600E* may lose statistical significance after multivariate adjustment [26,62]. Previous meta-analyses have reported inconsistent estimates of the prognostic effect of *BRAF V600E*, in part because of differences in endpoint definitions, pooling strategies, and adjustment structure. Gatta et al. [28] reported an association between *BRAF V600E* and recurrence in PTC, but that analysis incorporated broader outcome heterogeneity and did not prioritize tumor size-adjusted recurrence models in the way performed here. By contrast, our study was restricted to multivariate-adjusted hazard ratios and further tested robustness through outcome-stratified analysis. These data indicate that the prognostic effect of *BRAF V600E* is present but modest, with the strongest association observed for recurrence-related outcomes rather than for disease-free survival overall. Two other meta-analyses by Li et al. [25] and Wang et al. [8] failed to demonstrate an association between *BRAF V600E* mutation and recurrence in PTC. These discrepancies are likely explained by differences in study selection, endpoint definitions, and the extent of multivariable adjustment across the included cohorts. By synthesizing only adjusted hazard ratios, our study suggests that *BRAF V600E* provides modest complementary prognostic information beyond established clinicopathologic factors—a risk signal that does not supplant the central role of tumor size and nodal burden. Beyond prognostication, *BRAF V600E* has therapeutic significance because combined *BRAF* and *MEK* inhibition with dabrafenib and trametinib has been approved for selected patients with *BRAF V600E*-mutant solid tumors, including advanced thyroid cancer settings [63].

The present analysis also illustrates the importance of methodological rigor in prognostic meta-analyses. Pooling unadjusted effect estimates may exaggerate the apparent impact of biomarkers that are closely associated with adverse clinicopathologic characteristics. By restricting the primary analysis to multivariable-adjusted hazard ratios and prioritizing studies that adjusted for tumor size or T category, our objective was not to maximize the observed effect size but to obtain a more clinically meaningful estimate of the independent association between *BRAF V600E* and recurrence. This approach differs from analyses that combine adjusted and unadjusted estimates and therefore provides a more conservative assessment of the prognostic contribution attributable to the mutation itself.

Moderate between-study heterogeneity was observed (I^2^ ≈ 56%), indicating that a substantial proportion of variability across studies reflects true differences rather than sampling error. Meta-regression analyses did not identify adjustment for tumor size or lymph node status as significant sources of heterogeneity, suggesting that other unmeasured factors—such as differences in treatment strategies, molecular characteristics, follow-up duration, or study design—may contribute to the observed variability. Although the direction of effect was generally consistent across studies, the moderate heterogeneity indicates variability in effect magnitude between cohorts. Therefore, the pooled estimate should be interpreted as an average association across heterogeneous observational studies rather than a uniform effect. Egger’s regression test did not demonstrate evidence of small-study effects. However, this test evaluates publication bias rather than the overall credibility or validity of the pooled evidence. Residual heterogeneity likely reflects differences in surgical management, radioiodine treatment, duration of follow-up, molecular background, and recurrence definitions across the included studies.

Accordingly, current guidelines recommend cautious interpretation of *BRAF* mutation status and advise against altering management solely on the basis of mutation positivity [21]. Clinically, its utility may be greatest in patients classified at the borderline between low and intermediate risk—such as those with microscopic extrathyroidal extension or limited lymph node involvement—where modest increases in recurrence risk may justify closer surveillance or more individualized decision-making regarding adjuvant therapy. From a clinical perspective, these findings may have implications for postoperative surveillance rather than initial treatment selection. Patients with borderline clinicopathologic risk profiles may derive the greatest benefit from incorporating *BRAF V600E* into individualized follow-up strategies, whereas patients with clearly low-risk or high-risk disease are unlikely to have management substantially altered by *BRAF* status alone. Future prognostic models should therefore focus on integrating *BRAF V600E* with additional molecular markers, histopathologic variables, and dynamic risk stratification approaches to improve prediction of recurrence beyond what can be achieved by any single biomarker.

This study has several important limitations. First, the included studies used heterogeneous recurrence-related endpoints, including structural recurrence, recurrence-free survival, disease-free survival, persistent structural disease, and progression-free survival, which represent related but not identical clinical outcomes. Second, all included studies were retrospective observational cohorts, limiting causal inference. Third, inclusion was restricted to studies reporting multivariable-adjusted hazard ratios, which strengthened the evaluation of adjusted prognostic associations but may have excluded otherwise relevant cohorts that did not report time-to-event estimates in an extractable format. Finally, the multivariable adjustment strategies differed across studies, particularly with respect to lymph node status, extrathyroidal extension, age, and other clinicopathologic covariates, which may have contributed to between-study heterogeneity. Additionally, molecular profiling was incomplete in nearly all cohorts, preventing evaluation of known synergistic oncogenic interactions—particularly *BRAF V600E* with *TERT* promoter mutations. Previous large cohort studies have demonstrated that this molecular combination identifies a distinct high-risk subgroup of PTC characterized by increased recurrence rates, resistance to radioiodine therapy, and poorer survival outcomes [4,64]. These observations suggest that the clinical utility of *BRAF* may be substantially enhanced when interpreted in conjunction with additional molecular markers, particularly *TERT* promoter status. Future studies incorporating integrated molecular profiling are therefore essential to refine risk stratification and to identify patients who may benefit from more aggressive initial management or closer postoperative surveillance. Although funnel plots did not reveal major publication bias, subtle reporting bias cannot be fully excluded. Inter-rater agreement was not formally quantified using kappa statistics due to the retrospective nature of the review process, representing a methodological limitation. Another limitation was that prospective protocol registration (e.g., PROSPERO) was not performed. Finally, because all included studies were observational, residual confounding cannot be completely excluded despite multivariable adjustment.

## 5. Conclusions

In conclusion, this meta-analysis demonstrates that *BRAF V600E* is associated with a modest but statistically significant increase in recurrence risk in PTC, particularly for structural recurrence and recurrence-related outcomes. These findings support considering *BRAF V600E* as a complementary component of postoperative risk assessment. Clinical decisions should continue to be guided primarily by established clinicopathologic factors, with *BRAF* interpreted within a broader risk-stratification framework. Future efforts should focus on large, prospectively followed cohorts with comprehensive molecular annotation to refine individualized recurrence prediction and define scenarios in which *BRAF* status meaningfully influences clinical decision-making.

## Figures and Tables

**Table 1:** Characteristics of included studies.

S. No.	First Author	Year	Country	Study Type	Total Sample Size	*BRAF* Positive	*BRAF* Negative	Outcome Reported
1	Park KW [30]	2026	South Korea	Retrospective	471	398	73	Recurrence-free survival
2	Brumfield A [31]	2025	USA	Retrospective	301	237	64	Recurrence
3	Radi S [32]	2025	Canada	Retrospective	50	26	24	Disease-free survival
4	Han S [33]	2025	China	Retrospective	1118	700	418	Recurrence
5	Nguyen VPT [20]	2025	Japan	Retrospective	603	476	127	Disease-free survival
6	Gil-Bernabé S [34]	2025	Spain	Retrospective	41	22	19	Recurrence
7	Yang F [35]	2023	China	Retrospective	374	249	125	Recurrence-free survival
8	Issa PP [24]	2023	USA	Retrospective	469	148	321	Recurrence
9	Parvathareddy SK [36]	2022	Saudi Arabia	Retrospective	1640	910	730	Disease-free survival
10	Zurnadzhy L [37]	2022	Ukraine	Retrospective	428	136	292	Disease-free survival
11	Xiang Y [38]	2021	China	Retrospective	182	73	109	Recurrence
12	Fang H [39]	2021	China	Retrospective	57	32	25	Recurrence
13	Enumah S [40]	2020	USA	Retrospective	411	260	151	Recurrence
14	Capdevila J [41]	2020	Spain	Retrospective	125	33	92	Progression-free survival
15	Gan X [42]	2020	China	Retrospective	475	239	236	Recurrence
16	Pamedytyte D [43]	2020	Lithuania	Retrospective	205	127	78	Disease-free survival
17	Camargo Barros-Filho M [44]	2019	Brazil	Retrospective	112	68	44	Recurrence
18	Huang Y [45]	2018	USA, Italy, Poland, Australia, Spain, Czech Republic	Retrospective	955	321	634	Recurrence
19	Shen X [46]	2017	USA	Retrospective	388	226	162	Recurrence
20	Kim Y [47]	2017	Korea	Retrospective	428	353	75	Disease-free survival
21	Takacsova E [48]	2017	Slovakia	Retrospective	199	103	96	Disease-free survival
22	Lee SE [49]	2016	Korea	Retrospective	237	177	60	Recurrence
23	Fraser S [50]	2016	Australia	Retrospective	496	309	187	Recurrence
24	Xing M [19]	2015	USA	Retrospective	2099	1017	1082	Recurrence
25	Xing M [4]	2014	USA	Retrospective	507	194	313	Recurrence
26	Russo M [51]	2014	Italy	Retrospective	103	57	46	Recurrence/Persistence
27	Yim JH [52]	2014	South Korea	Retrospective	164	123	41	Recurrence
28	Fernandez IJ [53]	2013	Italy	Retrospective	297	173	124	Recurrence-free survival
29	Zheng X [54]	2013	China	Retrospective	977	392	585	Recurrence
30	Gong RX [55]	2013	China	Retrospective	187	119	68	Recurrence
31	Prescott JD [56]	2012	USA	Retrospective	205	110	95	Recurrence
32	Ulisse S [57]	2012	Italy	Retrospective	91	44	47	Recurrence
33	Stanojevic B [58]	2011	Serbia	Retrospective	266	84	182	Disease-free survival
34	Elisei R [59]	2008	Italy	Retrospective	102	38	64	Disease-free survival
35	Xing M [60]	2005	USA, Ukraine, Italy	Retrospective	219	107	112	Recurrence

## Data Availability

The data presented in this study are available in this article.

## Supplementary Materials

The following supporting information can be downloaded at https://www.mdpi.com/article/10.3390/cancers18142299/s1, Supplementary Figure S1: Forest plot (subgroup analysis): Forest plot showing the association between *BRAF V600E* mutation and recurrence among 26 studies that reported recurrence-related outcomes; Supplementary Figure S2: Forest plot (subgroup analysis): Forest plot showing the association between *BRAF V600E* mutation and disease-free survival among nine studies that reported disease-free survival; Supplementary Figure S3: Trim-and-fill analysis. The closed dots indicate the observed studies, and the open dots indicate the missing studies imputed by the trim-and-fill method. One potentially missing study on the left side of the funnel plot was imputed; Supplementary Table S1: PRISMA 2020 Checklist; Supplementary Table S2: Summary of adjusted variables and Newcastle–Ottawa Scale score for each included study.
