# Supplementary material for: Independent Prognostic Value of BRAF V600E for Recurrence in Papillary Thyroid Carcinoma: A Systematic Review and Meta-Analysis
Source: Cancers (Basel). 2026 Jul 17;18(14):2299. doi: 10.3390/cancers18142299 (PMC13406699; doi:10.3390/cancers18142299)
Supplement: Supplementary file 1 [file cancers-18-02299-s001.zip › cancers-4410873-supplementary.pdf]

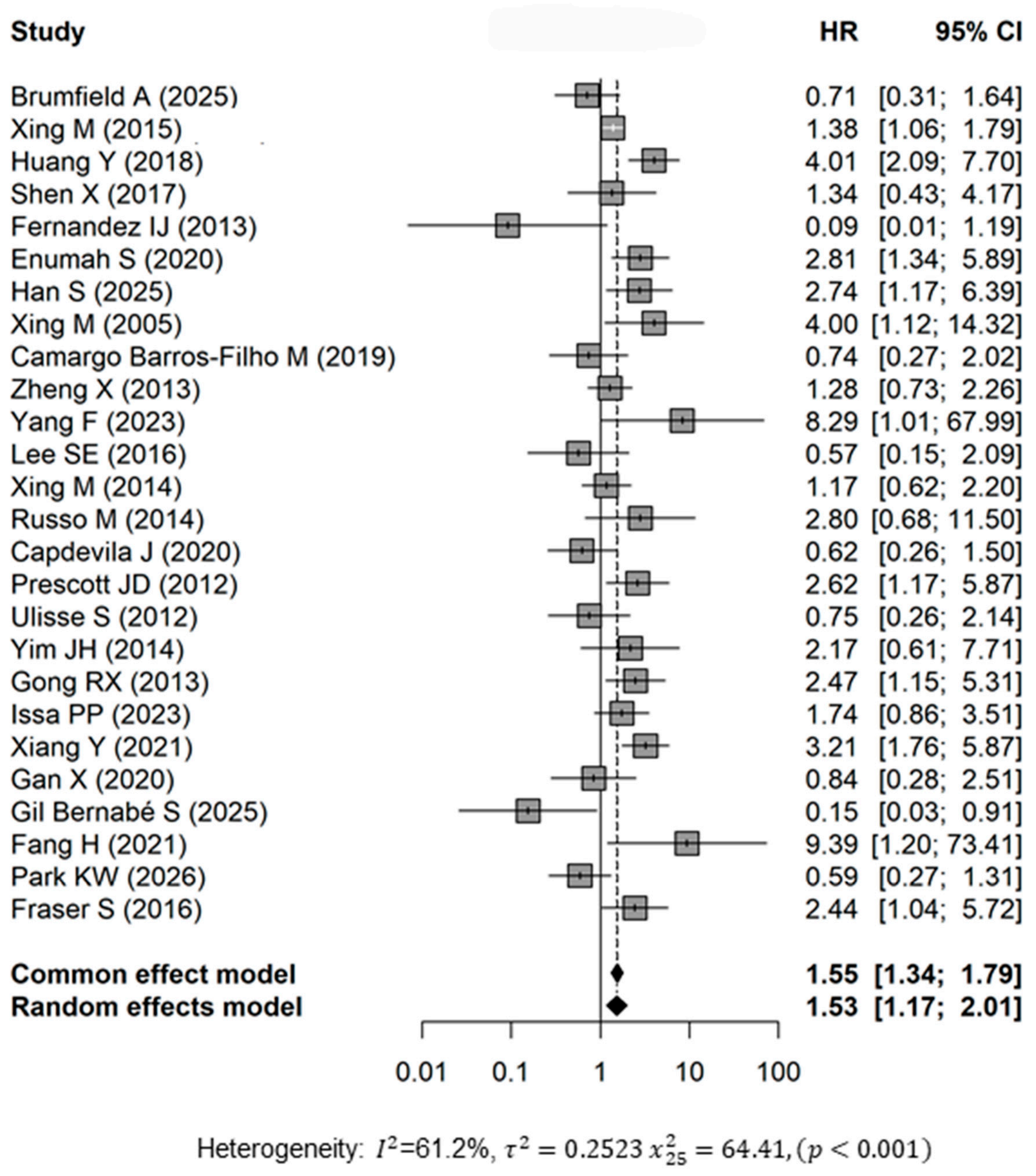

Supplementary Figure S1: Forest plot (subgroup analysis): Forest plot showing the association between *BRAF V600E* mutation and recurrence among 26 studies ([31], [19], [45], [46], [53], [40], [33], [60], [44], [54], [35], [49], [4], [51], [41], [56], [57], [52], [55], [24], [38], [42], [34], [39], [30] and [50], respectively) that reported recurrence-related outcomes.

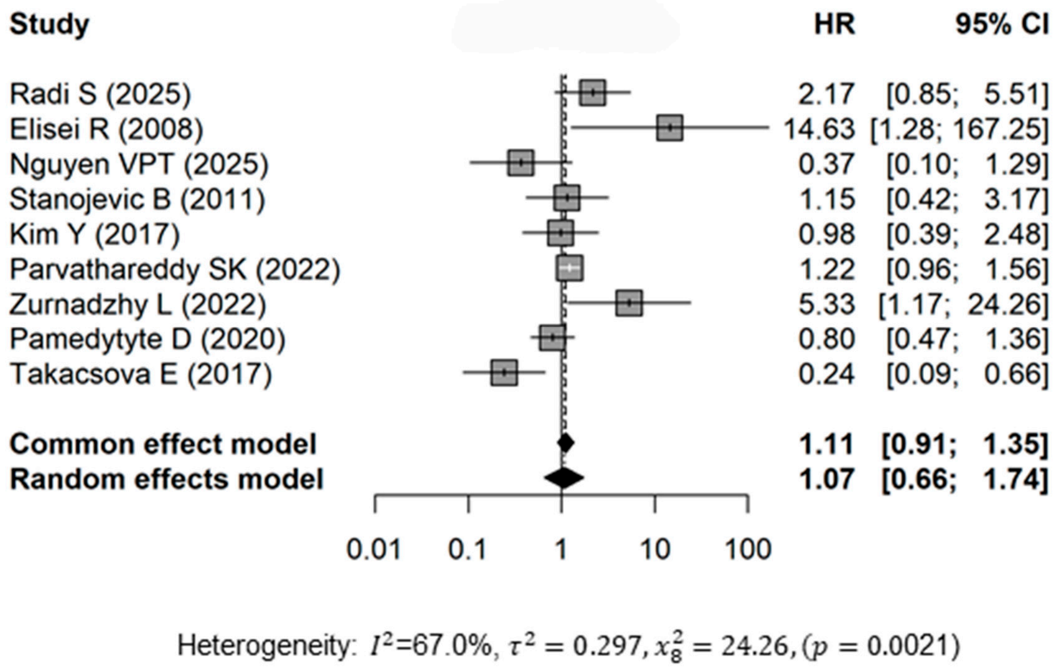

Supplementary Figure S2: Forest plot (subgroup analysis): Forest plot showing the association between *BRAF V600E* mutation and disease-free survival among nine studies ([32], [59], [20], [58], [47], [36], [37], [43] and [48], respectively) that reported disease-free survival.

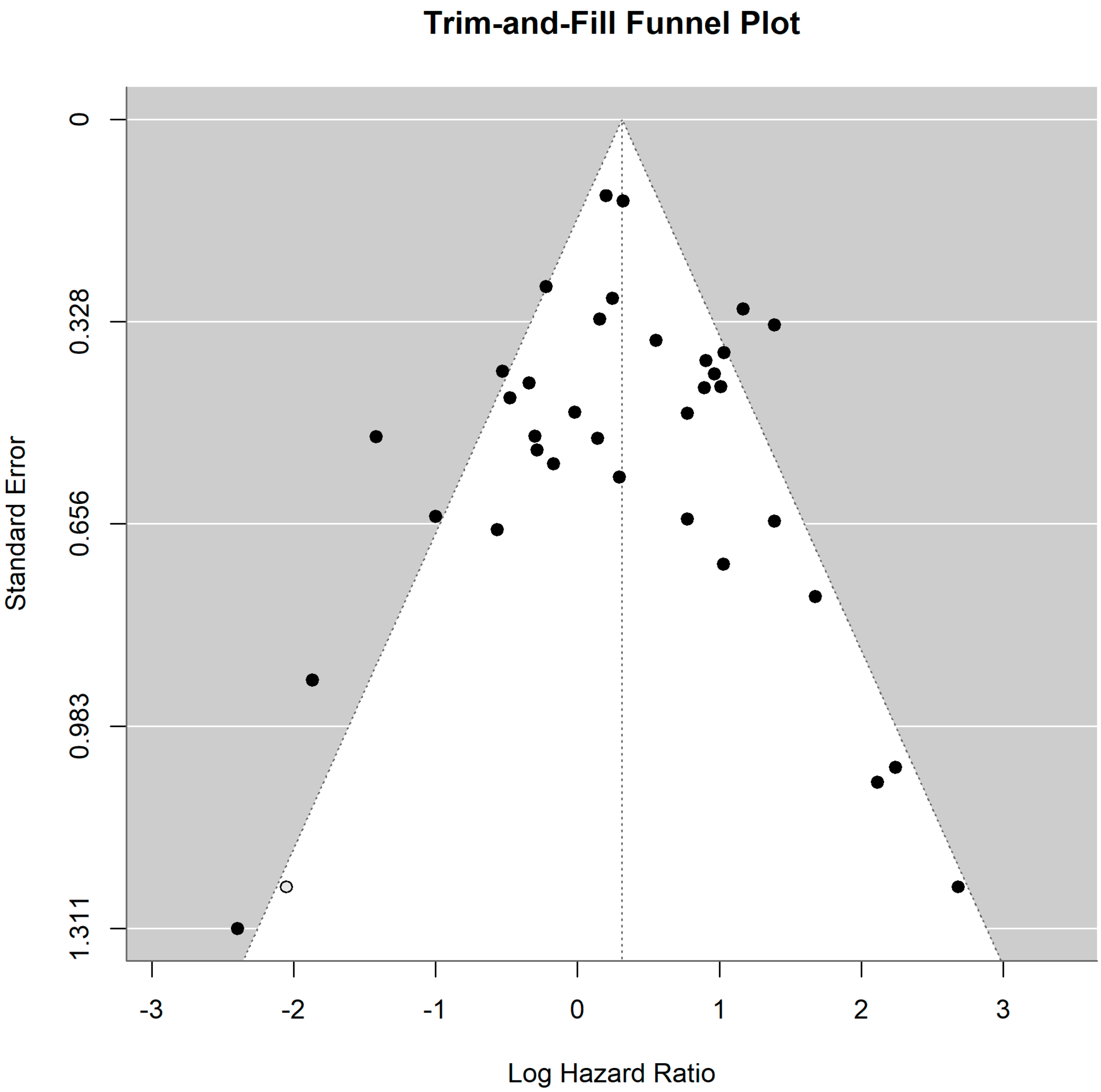

Supplementary Figure S3: Trim-and-fill analysis. The closed dots indicate the observed studies, and the open dots indicate the missing studies imputed by the trim-and-fill method. One potentially missing study on the left side of the funnel plot was imputed.

Supplementary Table S1. PRISMA 2020 Checklist

Section and Topic	Item #	Checklist item	Location where item is reported
**TITLE**			
Title	1	Identify the report as a systematic review.	Page no. 1
**ABSTRACT**			
Abstract	2	See the PRISMA 2020 for Abstracts checklist.	Page no. 1
**INTRODUCTION**			
Rationale	3	Describe the rationale for the review in the context of existing knowledge.	Page no. 3
Objectives	4	Provide an explicit statement of the objective(s) or question(s) the review addresses.	Page no. 3
**METHODS**			
Eligibility criteria	5	Specify the inclusion and exclusion criteria for the review and how studies were grouped for the syntheses.	Page no. 4
Information sources	6	Specify all databases, registers, websites, organisations, reference lists and other sources searched or consulted to identify studies. Specify the date when each source was last searched or consulted.	Page no. 3
Search strategy	7	Present the full search strategies for all databases, registers and websites, including any filters and limits used.	Page no. 3
Selection process	8	Specify the methods used to decide whether a study met the inclusion criteria of the review, including how many reviewers screened each record and each report retrieved, whether they worked independently, and if applicable, details of automation tools used in the process.	Page no. 4
Data collection process	9	Specify the methods used to collect data from reports, including how many reviewers collected data from each report, whether they worked independently, any processes for obtaining or confirming data from study investigators, and if applicable, details of automation tools used in the process.	Page no. 4
Data items	10a	List and define all outcomes for which data were sought. Specify whether all results that were compatible with each outcome domain in each study were sought (e.g. for all measures, time points, analyses), and if not, the methods used to decide which results to collect.	Page no. 5
	10b	List and define all other variables for which data were sought (e.g. participant and intervention characteristics, funding sources). Describe any assumptions made about any missing or unclear information.	Page no. 4
Study risk of bias assessment	11	Specify the methods used to assess risk of bias in the included studies, including details of the tool(s) used, how many reviewers assessed each study and whether they worked independently, and if applicable, details of automation tools used in the process.	Page no. 4
Effect measures	12	Specify for each outcome the effect measure(s) (e.g. risk ratio, mean difference) used in the synthesis or presentation of results.	Page no. 4
Synthesis methods	13a	Describe the processes used to decide which studies were eligible for each synthesis (e.g. tabulating the study intervention characteristics and comparing against the planned groups for each synthesis (item #5)).	Page no. 4

Section and Topic	Item #	Checklist item	Location where item is reported
	13b	Describe any methods required to prepare the data for presentation or synthesis, such as handling of missing summary statistics, or data conversions.	Page no. 4
	13c	Describe any methods used to tabulate or visually display results of individual studies and syntheses.	Page no. 4
	13d	Describe any methods used to synthesize results and provide a rationale for the choice(s). If meta-analysis was performed, describe the model(s), method(s) to identify the presence and extent of statistical heterogeneity, and software package(s) used.	Page no. 4
	13e	Describe any methods used to explore possible causes of heterogeneity among study results (e.g. subgroup analysis, meta-regression).	Page no. 4
	13f	Describe any sensitivity analyses conducted to assess robustness of the synthesized results.	Page no. 4
Reporting bias assessment	14	Describe any methods used to assess risk of bias due to missing results in a synthesis (arising from reporting biases).	Page no. 4
Certainty assessment	15	Describe any methods used to assess certainty (or confidence) in the body of evidence for an outcome.	Page no. 4
**RESULTS**			
Study selection	16a	Describe the results of the search and selection process, from the number of records identified in the search to the number of studies included in the review, ideally using a flow diagram.	Page no. 5-6
	16b	Cite studies that might appear to meet the inclusion criteria, but which were excluded, and explain why they were excluded.	Page no. 6
Study characteristics	17	Cite each included study and present its characteristics.	Page no. 6-7
Risk of bias in studies	18	Present assessments of risk of bias for each included study.	Page no. 10
Results of individual studies	19	For all outcomes, present, for each study: (a) summary statistics for each group (where appropriate) and (b) an effect estimate and its precision (e.g. confidence/credible interval), ideally using structured tables or plots.	Page no. 7-9
Results of syntheses	20a	For each synthesis, briefly summarise the characteristics and risk of bias among contributing studies.	Page no. 10
	20b	Present results of all statistical syntheses conducted. If meta-analysis was done, present for each the summary estimate and its precision (e.g. confidence/credible interval) and measures of statistical heterogeneity. If comparing groups, describe the direction of the effect.	Page no. 9-10
	20c	Present results of all investigations of possible causes of heterogeneity among study results.	Page no. 10
	20d	Present results of all sensitivity analyses conducted to assess the robustness of the synthesized results.	Page no. 8
Reporting biases	21	Present assessments of risk of bias due to missing results (arising from reporting biases) for each synthesis assessed.	Page no. 10
Certainty of evidence	22	Present assessments of certainty (or confidence) in the body of evidence for each outcome assessed.	Page no. 10

Section and Topic	Item #	Checklist item	Location where item is reported
**DISCUSSION**			
Discussion	23a	Provide a general interpretation of the results in the context of other evidence.	Page no. 10-11
	23b	Discuss any limitations of the evidence included in the review.	Page no. 12
	23c	Discuss any limitations of the review processes used.	Page no. 12
	23d	Discuss implications of the results for practice, policy, and future research.	Page no. 12-13
**OTHER INFORMATION**			
Registration and protocol	24a	Provide registration information for the review, including register name and registration number, or state that the review was not registered.	Page no. 3
	24b	Indicate where the review protocol can be accessed, or state that a protocol was not prepared.	Page no. 3
	24c	Describe and explain any amendments to information provided at registration or in the protocol.	Page no. 3
Support	25	Describe sources of financial or non-financial support for the review, and the role of the funders or sponsors in the review.	Page no. 13
Competing interests	26	Declare any competing interests of review authors.	Page no. 13
Availability of data, code and other materials	27	Report which of the following are publicly available and where they can be found: template data collection forms; data extracted from included studies; data used for all analyses; analytic code; any other materials used in the review.	Page no. 13

Supplementary Table S2. Summary of adjusted variables and Newcastle Ottawa Scale score for each included study

S. No.	First Author	Year	Tumor size/T category adjustment	Age adjustment	Lymph node adjustment	Extrathyroidal extension adjustment	Radioiodine adjustment	Other covariates included	Follow-up duration (median)	Outcome reported	Newcastle-Ottawa Scale (NOS) Score
1	Park KW	2026	Yes	No	Yes	No	No	Sex, histologic subtype, SPHK1 expression	Not mentioned	Recurrence-free survival	9
2	Brumfield A	2025	No	No	Yes	Yes	No	Sex, histologic subtype, multifocality	23.4 months	Recurrence	8
3	Radi S	2025	Yes	Yes	No	Yes	No	Histologic subtypes, sex, Ki-67, lymphovascular invasion	36 months	Disease-free survival	9
4	Han S	2025	Yes	No	Yes	No	Yes	Sex, multifocality, type of surgery, local invasion	47 months	Recurrence	9
5	Nguyen VPT	2025	Yes	Yes	Yes	Yes	No	Sex, TERT promoter mutation	58 months	Disease-free survival	9
6	Gil-Bernabé S	2025	No	Yes	Yes	No	No	TERT amplification, sex, vascular invasion, Foci infiltrative insular tumor cells at advancing edge of tumor, Focal tall cell appearance	150 months	Recurrence	9
7	Yang F	2023	No	No	Yes	No	No	Esophageal invasion, laryngotracheal invasion	57.7 months	Recurrence-free survival	9
8	Issa PP	2023	Yes	Yes	Yes	Yes	No	Sex, race, multifocality, capsular invasion, angioinvasion, laterality, Hashimoto's thyroiditis	46.1 months	Recurrence	9
9	Parvathareddy SK	2022	Yes	Yes	Yes	Yes	No	Sex, laterality, focality, distant metastasis	90 months	Disease-free survival	9
10	Zurnadzhy L	2022	Yes	No	Yes	Yes	Yes	Sex, multifocality, lymphovascular invasion, distant metastasis, type of surgery	Not mentioned	Disease-free survival	9
11	Xiang Y	2021	No	No	Yes	No	No	TSH level	60.8 months	Recurrence	8
12	Fang H	2021	Yes	No	No	No	No	Sex, histologic subtype, background lymphocytic infiltration	39 months	Recurrence	7
13	Enumah S	2020	Yes	Yes	Yes	Yes	Yes	Not reported	123.6 months	Recurrence	9
14	Capdevila J	2020	No	Yes	No	No	No	Sex, histologic subtype, sorafenib treatment, RAS mutation	Not mentioned	Progression-free survival	9
15	Gan X	2020	Yes	No	Yes	Yes	No	Sex,histologic subtype, multifocality, residual tumor, distant metastasis, stage	20 months	Recurrence	8
16	Pamedytyte D	2020	Yes	Yes	Yes	No	No	Sex, miR- 146b, miR-222, miR-21, miR-221, miR-181b	Not mentioned	Disease-free survival	9
17	Camargo Barros-Filho M	2019	No	No	Yes	Yes	No	Sex, multifocality, ATA risk, PFKFB2 Methylation	127 months	Recurrence	9
18	Huang Y	2018	Yes	Yes	No	No	No	Sex	64 months	Recurrence	9
19	Shen X	2017	Yes	Yes	Yes	Yes	No	Sex, multifocality,medical centre	Not mentioned	Recurrence	9
20	Kim Y	2017	Yes	Yes	Yes	No	No	Sex, aggressive variant, dyscohesive cells, psammoma bodies	70 months	Disease-free survival	9
21	Takacsova E	2017	No	Yes	No	No	No	Sex, risk categories	43 months	Disease-free survival	8
22	Lee SE	2016	Yes	Yes	Yes	Yes	No	TERT C228T status	48 months	Recurrence	9
23	Fraser S	2016	Yes	Yes	Yes	Yes	No	Sex	56.8 months	Recurrence	9
24	Xing M (2015)	2015	Yes	Yes	Yes	Yes	No	Sex, histologic subtype, multifocality	36 months	Recurrence	9
25	Xing M (2014)	2014	Yes	Yes	Yes	Yes	No	Sex, multifocality, vascular invasion	24 months	Recurrence	9
26	Russo M	2014	Yes	Yes	Yes	Yes	No	Sex, multifocality, stage	55 months	Recurrence/Persistence	9
27	Yim JH	2014	Yes	Yes	Yes	Yes	No	Sex, multifocality	132 months	Recurrence	9
28	Fernandez IJ	2013	Yes	No	Yes	No	No	AJCC stage, BMI, Tall cell, ATA risk	49.8 months	Recurrence-free survival	9
29	Zheng X	2013	Yes	Yes	Yes	Yes	No	Sex, multifocality, type of surgery	93.6 months	Recurrence	9
30	Gong RX	2013	Yes	No	Yes	No	No	Not reported	Not mentioned	Recurrence	9
31	Prescott JD	2012	Yes	No	Yes	No	No	Sex, multifocality, lymphovascular invasion, histologic subtype	Not mentioned	Recurrence	9
32	Ulisse S	2012	No	Yes	No	No	No	Sex, stage, urokinase plasminogen activator	64 months	Recurrence	9
33	Stanojevic B	2011	Yes	Yes	Yes	No	No	Not reported	53.1 months	Disease-free survival	9
34	Elisei R	2008	Yes	Yes	Yes	No	No	De Groot's classes, Stage, VEGF, vascular invasion	180 months	Disease-free survival	9
35	Xing M (2005)	2005	Yes	Yes	Yes	Yes	Yes	Sex, multifocality, stage, histologic subtype	15 months	Recurrence	9
